# Migration, perceived discrimination and the development of psychosis

**DOI:** 10.1192/j.eurpsy.2023.1744

**Published:** 2023-07-19

**Authors:** V. Barata, J. Bastos, C. Cativo, P. Gonçalves

**Affiliations:** Psychiatry, Hospital Prof. Dr. Fernando Fonseca, Lisboa, Portugal

## Abstract

**Introduction:**

Migration is a rapidly growing phenomenon in European countries and its association with psychotic disorders is a public health concern. Psychosis is more prevalent among migrants, which suggests that adverse social experiences play an important role in its pathogenesis. Throughout the migration process, migrants are exposed to several social disadvantages, including in the post-immigration context, where perceived discrimination appears to be an important stressor. In fact, the highest incidence rates of psychosis occur in the most discriminated populations, namely migrants with darker skin complexion, particularly when living in low-ethnic-density neighborhoods, where both discrimination and social isolation are more prominent.

**Objectives:**

To conduct an updated review about the association between migration, perceived discrimination and psychosis, aiming to better understand the mechanisms involved.

**Methods:**

Narrative literature review using the keywords “migration”; “psychosis”; “discrimination”; “racism” on PubMed database, in conjunction with presentation of a clinical case concerning a patient from Guinea-Bissau, admitted to our hospital in the context of first-episode psychosis (FEP), with onset months after completing the Mediterranean migration route to Europe.

**Results:**

Literature suggests that experiences of racism and social exclusion contribute to feelings of imminent danger, fear and general anxiety, which may develop into paranoid ideas of ubiquitous persecution. Furthermore, intense social defeat experiences, common in migrants, are associated with more distressing forms of delusional content, with delusions of psychological persecution being more common. However, there is also evidence that migrants with FEP have better occupational and social functioning profiles compared to natives, suggesting that, in these patients, there is a higher burden of social-environmental risk factors, with the onset of psychosis occurring only when this burden overcomes a higher threshold. Our patient fits this description. After completing his migratory route and while living in an Italian refugee camp, he described suffering experiences of severe discrimination. Real or not, these experiences escalated to become delusional ideas of persecution involving European governments, thought to seek for his humiliation. Despite the presence of psychotic symptoms, this patient was able to maintain a reasonable level of functioning during years, up to his psychiatric admission.

**Conclusions:**

Given the notorious effect of perceived discrimination and racism on the increased risk of psychosis in immigrants, it is urgent to adopt policies that promote the social protection of these vulnerable groups, namely through enhancing their integration in the host countries.

**Disclosure of Interest:**

None Declared

